# 2309. Ceftaroline for Central Nervous System and Ocular Infections

**DOI:** 10.1093/ofid/ofac492.141

**Published:** 2022-12-15

**Authors:** Emily A Siegrist, Joseph Sassine

**Affiliations:** OU Health, Oklahoma City, Oklahoma; University of Oklahoma Health Sciences Center, Oklahoma City, Oklahoma

## Abstract

**Background:**

Ceftaroline is increasingly used in combination therapy for methicillin resistant *Staphylococcus aureus* (MRSA) bacteremia. Animal models suggest ceftaroline penetrates the central nervous system (CNS) and vitreous humor; however, there are limited data which describe outcomes of patients treated with ceftaroline for infections with CNS or ocular involvement.

**Methods:**

This was a single-center, retrospective study at an academic medical center. The primary objective was to describe outcomes in patients treated with ceftaroline for CNS or ocular infections. Patients who received ceftaroline from January 2015 to February 2022 were identified. Patients > 18 years who received > 48 hours of ceftaroline and had evidence of CNS or ocular involvement were included. Descriptive statistics were used.

**Results:**

Ten patients met the inclusion criteria: six with CNS and four with ocular infections. The cohort was predominantly white (90%) and male (70%). The most common comorbidities were hypertension (70%) and diabetes (40%) (Table 1). Nine patients were treated for MRSA and one patient was treated for methicillin resistant *Staphylococcus epidermidis* (Table 2). Ceftaroline was salvage therapy in nine patients (90%), often following vancomycin and used as part of combination therapy (Table 3).

Of the four patients with ocular infections, all had MRSA bacteremia. Two patients (50%) had positive vitreal cultures and received intra-vitreal injections (Table 2). Most of these patients (75%) died during admission (Table 4). Of the six patients with CNS infections, injection drug use was more common than in patients with ocular involvement (33% vs 0%), as was immunocompromise (50% vs 0%) and the presence of endocarditis (33% vs 0%). Of the patients with CNS involvement, three had abnormal lumbar punctures and the remainder were diagnosed by CNS imaging (Table 2). Four patients with CNS involvement survived hospital stay (67%), and one of these had recurrence of CNS infection within 90 days (Table 4).

**Conclusion:**

Although patients with CNS or ocular involvement represent a severely ill subset of staphylococcal infections, ceftaroline is a promising agent in combination therapy. Comparative data is needed to validate these findings.

**Disclosures:**

**All Authors**: No reported disclosures.

